# ‘White Child Gone Bankrupt’—The Intersection of Race and Poverty in Youth Fathered by UN Peacekeepers

**DOI:** 10.1007/s11013-022-09772-7

**Published:** 2022-03-18

**Authors:** Kirstin Wagner, Susan A. Bartels, Sanne Weber, Sabine Lee

**Affiliations:** 1 Department of History, University of Birmingham, Birmingham, B15 2TT UK; 2 Department of Emergency Medicine and Public Health Sciences, Queen’s University, 18 Barrie Street, Kingston, K7L 3N6 Canada; 3 International Development Department, University of Birmingham, Birmingham, B15 2TT UK

**Keywords:** Social identity, Peacekeeping, Children, Poverty, Race

## Abstract

Children fathered and abandoned by United Nations peacekeepers are an unintended consequence of peacekeeping operations. Research suggests that the social identity of peacekeeper-fathered children (PKFC) is complex and contradictory. While economically disadvantaged, PKFC’s biracial background confers elements of racial privilege. Using the Democratic Republic of Congo as a case study, the present research evaluates the impact of racial differences on PKFC’s social standing. Drawing on in-depth interviews with a racially heterogeneous sample of 35 PKFC and 60 mothers, we analyse how race and poverty interact and cause PKFC’s conflicting social role. The data demonstrates that being of mixed race leads to the expectation of a higher living standard. Since most PKFC live in extreme economic deprivation, their anticipated privilege contrasts with reality. We found that the stigmatizing effects of poverty were amplified by biracial identification, leading to additional disadvantage, epitomised in the term “Muzungu aliye homba” [white child gone bankrupt]. The findings add to research on ‘children born of war’ and show the role of culture in shaping youth’s social identities. Based on PKFC’s intersecting burdens, we make policy recommendations that address the nexus of race and poverty.

## Introduction

My friends say that all of us are children but not in the same way. They say that we are different. I don’t want them to talk like that. I don’t feel happy then. (PKFC)

Sexual and gender-based violence perpetrated by military, police and civilian personnel associated with peacekeeping operations is defined by the United Nations (UN) as sexual exploitation and abuse (SEA) (UN General Assembly 2003). The lack of resources and security in many conflict settings makes civilians susceptible to SEA and allows sex trade and sex trafficking to flourish when missions are deployed (Beber et al. 2017). This has been attributed to the fact that civilians who live in conditions of abject poverty and social unrest, engage in sexual relations with peacekeepers as a strategy to improve their dire financial situations and gain safety and protection (Utas 2005; Westendorf and Searle 2017). In spite of the UN’s persistent efforts to eliminate sexual relations between peacekeeping personnel and local populations, the continuation of such relations is evidenced by the growing number of mixed-race children in host state communities (Lee and Bartels 2019; Harrington 2007). The Democratic Republic of Congo (DRC), in particular, has emerged as the setting for mounting reports of male peacekeepers implicated in rape, transactional sex, and sexually exploitative relationships leading to pregnancy and childbirth (Wagner et al. 2020; Notar 2006; UN General Assembly 2007). Although it is acknowledged that peacekeeper-fathered children (PKFC) are an unintended legacy of peacekeeping missions, relatively little has been done to establish an evidence base relating to their life courses.

Since the early 2000s, claims that members of UN peacekeeping forces are fathering children in the fragile settings where they operate have been a matter of concern for public and scholarly debate (Lynch 2005; Ndulo 2009). According to media reports, mothers of PKFC are “extremely impoverished and face discrimination, stigma and the heartache of being abandoned to look after their children alone” (McVeigh 2017). Early empirical evidence confirms that the absence of peacekeeper fathers often leaves mothers to raise their children in extreme socio-economic deprivation (Lee and Bartels 2019, Vahedi et al. 2020). Research into the situation of PKFC identified multiple areas of disadvantage, most importantly their lack of support from peacekeeper fathers, the UN, or family and kin networks (Wagner et al. 2020, 2022). However, focusing solely on their economic needs can obscure the complex reality of their existence.

Despite being disadvantaged, scholars have noted that the mixed-race background of PKFC might confer elements of racial privilege, leading to a more diverse and contradictory social standing. Higate and Henry (2004:492) indicated that the multi-faceted impact of PKFC on communities could include an elevation in social status where being of mixed race is interpreted as desirable or “racially superior”. Fathered by UN personnel from troop-contributing countries around the world, PKFC often hold features that clearly identify them as being of different ethnicity, making them stand out as biracial (Lee 2017; Ndulo 2009). In Haiti, women were sometimes perceived to be seeking out relationships with peacekeepers “with the goal to have beautiful, mixed-race children” (Lee and Bartels 2019:197). Thus, the cultural and societal perceptions of race in host state communities hold implications for how PKFC are situated in society. Koyama and Myrttinen (2007) found that in Timor-Leste, PKFC were treated differently based on their fathers’ sending state—an indication that their life experiences as biracial children are qualitatively distinct not just from non-PKFC but also from one another.

To date, no academic, news or policy report has addressed the racial background of PKFC specifically or examined their presumed racial privilege. The present research is the first to evaluate the importance of their racial heritage and reflect on PKFC as a racially heterogeneous group. By considering the interaction of mixed race and poverty, the article is concerned with how PKFC’s differences determine their social relations and lead to an increased or reduced risk for stigmatisation. According to the literature, PKFC might be perceived as having a dominant racial identity that is interpreted as constituting a privileged social status. Due to their economic deprivation, they might belong to a lower social class and receive an inferior education than their peers, providing them with fewer opportunities. We will discuss whether the interaction of these factors constitutes a conflicting social role and evaluate how racial differences contribute to a more or less privileged social identity. In doing so, we break away from simplistic explanations of PKFC as a uniform group but consider the roots of their outsider status. Using the UN Stabilization Organization Mission in eastern DRC as a case study, we will discuss how being of mixed race is viewed in an otherwise monoracial post-colonial setting and address the social, cultural and political parameters of racial identity in this context. In this way, the article contributes to understanding childhood in the socio-political and cultural context of armed conflict, and to explore how children’s exposure to poverty and race-based social hierarchies impacts their identity and self-perception.

The article employs insights from women’s studies, ethnic studies, psychology, and history to analyze the social position of PKFC in the wake of colonial influences, gender and racial oppression. In order to conceptualize how social status is construed and interpreted in the African context, we will first discuss how societies are organized using an intersectional approach to social identity.

## Theoretical Background

Linking the self to society, intersectionality theory (Crenshaw 1989, 1991) addresses the interactivity of social identity structures like race with gender and other factors that have historically fostered inequality. The concept of intersectionality emphasizes the multi-layered nature of social identity and promotes the awareness of asymmetrical relations between identity-forming experiences (Gopaldas 2013). Accordingly, personal identity reflects a combination of group memberships and social processes that create unique social positions for individuals. Identifying with multiple marginalized groups simultaneously can lead to unique forms of disadvantage, low self-esteem and multiple minority stress (Crenshaw 1989; Cyrus 2017). Although most research employing an intersectional lens focuses on underprivileged groups, intersectionality also helps understanding social advantages or conflicting social roles (Cole 2009). By investigating social identities as mutually constructed rather than additive (e.g., being a black woman vs being black and a woman), an intersectional analysis can be used to deconstruct binaries and analyze the variability within groups (McCall 2005).

Researchers applying the framework in social psychology emphasise how a sense of belonging to a privileged or oppressed group is formed, and explore how interrelating identities are combined (Settles and Buchanan 2014). According to social identity theory (Tajfel and Turner 1979), group memberships develop through a process of social comparison during which one categorises oneself as part of an in-group while others are referred to as the out-group (Stets and Burke 2000). The process of cross-categorization describes negotiations according to which individuals can be different along one set of criteria but similar along another (Deschamps and Doise 1978). An intersectional approach to social identity takes multiple social identities into account and examines how they are organised in a person’s self-concept (Freeman 2003). For biracial children, self-conception is considerably more challenging as they need to integrate their dual racial and/or cultural identification into all the other identity-forming experiences growing up (Lichter and Qian 2018).

### Race, Social Status and Children

The value children place on their racial identity is higher for racial-minority and mixed-race children than their majority peers (Turner and Brown 2007). A sense of mixed ethnicity has been found to evolve early in children in accordance with easily recognisable genetic and social markers such as appearance, language, class, religion or nationality (Goodman 1968; Rogers et al. 2012). By the age of five, most children can apply racial labels and show in-group bias towards their own racial group (Pfeifer et al. 2007; Slaughter-Defoe 2012). By mid-to-late childhood, awareness of race increases and children may perceive and enact racial stereotyping (Spears Brown and Bigler 2005).

High-status favouritism in children’s group evaluations indicates that they are aware of the association between race and social status (Bigler, Averhart and Liben 2003; Shutts et al. 2011). In South Africa, a country with a long history of race-based social inequality, children as young as four were found to associate higher status racial groups with higher levels of wealth (Olson et al. 2012). Olson et al. found a general tendency for early primary school children to match high value belongings with white people’s images over mixed-race or black people’s images. This shows that children are sensitive to social hierarchies and notice racial- and wealth-based inequalities from an early age. Studies from South Africa afford a unique opportunity to investigate children’s knowledge about racial groups in a racially diverse post-colonial setting and show that although blacks are more numerous than whites and hold significant power, pro-white bias can be observed in children (Olson et al. 2012; Shutts et al. 2011).

## Present Study

This research aims to deconstruct the complexity of PKFC’s social identities by evaluating the implication of their biracial provenance. By looking at the status differences of a racially heterogeneous sample of PKFC, we will assess whether mixed-race PKFC are afforded unique opportunities, as has sometimes been suggested in academic literature. In the process of evaluating their perception of identity, cultural and societal perceptions of race will be discussed against the backdrop of intersectionality and social identity theory. This will create an understanding of whether PKFC’s identify-forming experiences vary based on their fathers’ sending states. In analysing social identity through an intersectional lens, the article shows that PKFC are uniquely (dis)advantaged if white racial identity intersects with a relatively (low)high economic status.

Although racial categories and the related colour terms ‘white’, ‘brown’ and ‘black’ are controversial, they are critical key concepts emerging from the narratives of those participating in the present study and thus, will be applied as a locally significant source of classification. In order to acknowledge the interconnections between race and poverty, we will apply elements of an intersectional analysis. Since intersectionality is a signal contribution of feminist studies with an inherent focus on gender, we are not following a traditional intersectional approach, as we believe that intersections with gender will become more clearly reflected in older PKFC and should be the subject of future study.

In eastern DRC, where the study was conducted, racial inequality is a remnant of the colonial era. The DRC is a racially homogenous, predominantly black setting with beliefs about whiteness, white supremacy and black inferiority introduced by colonialism and reinforced by the global North’s role in recent conflicts (Jourdan 2013). Although ‘blackness’ operates as the normative foundation of race, social hierarchies with racialized dimensions, illustrated, for instance, in the white-dominated humanitarian and development industry, take a toll on the prospect of racial equality (Marchais, Bazuzi and Amani Lameke 2020). To justify a white civilization’s superiority, the Belgian colonial administrations created a race- and privilege-based social hierarchy with whites as the highest status group. The racist ideology of colonial order is exemplified in the abduction of mixed-race children (called ‘métis’, ‘mulattos’ or ‘children of sin’) who were born to African mothers between 1908 and 1960 and forcefully repatriated to Europe (Van Hooste 2020). Considered both a product of the colonial system, as well as a threat to it, the “métis problem” was defined by the question of whether children with mixed descent were to be classified as ‘white’ and thus, ‘superior’ in their ancestry or ‘black’ and ‘uncivilized’ (Van Hooste 2020:51). Reflecting the patriarchal biologies of colonial gender regimes, the colonial state feared the “négrification” of the European population and thus attempted to restrain African female sexuality and prevent interracial relations (Van Hooste 2020:15). This example of gendered racialization illustrates the European-introduced division of Congolese communities along racial, class and gender lines. Little is known about race relations in eastern DRC today, and no information exists on how mixed-race is perceived and interpreted. Belonging to the relatively small fair-skinned population within eastern DRC, the stories of PKFC offer a productive tool to research perceptions of race in this context.

## Methods

The research was conducted over three months, with 95 participants (60 mothers and 35 PKFC) as part of a larger study investigating peacekeeper-civilian interactions in eastern DRC in 2018. The objective of the here-analysed interviews was to determine through an exploratory-descriptive inquiry how mothers and PKFC experience the absence of peacekeeper fathers, their resulting life circumstances and social identities. Cole (2009) noted that in order to gain an understanding of social identity that is sensitive to group differences and similarities, an interpretative qualitative approach often leads to more meaningful results than a statistical analysis. Qualitative interviews were therefore used to emphasise inter-and intragroup variation in PKFC’s social identities.

We employed a conversational, semi-structured interview style with topic questions to guide the discussions and prompts that followed up on participants’ narratives (Sands 2004). All questions were open-ended and only explored issues further when participants seemed willing and comfortable to discuss them. PKFC aged 6 to 20 years were invited to participate if their mothers confirmed that they knew about the circumstances of their conception. The number of mothers in the study exceeds that of PKFC since some youth were excluded due to their age or unawareness of their family situation. While PKFC were asked to detail how they understood their social identity, mothers added information regarding their PKFC’s social identification, cultural norms and perceptions of race in the surrounding context.

The study was conducted in cooperation with Congolese community partners from Marakuja Kivu Research and Solidarité Féminine pour la Paix et le Développement Intégral (SOFEPADI). Both organisations were involved in the formation of interview guides and helped to implement the study. Two female social workers from SOFEPADI conducted the interviews that are analysed here. Both had experience in working with vulnerable populations and completed a five-day training prior to the start of the study. SOEFEPADI also set up a referral system for counselling and other service needs emerging from the interviews.

The fieldwork was located in urban centres in eastern DRC, namely Beni (North Kivu), Bukavu (South Kivu), Bunia (Ituri), Goma (North Kivu), Kalemie (Tanganyika) and Kisangani (Tshopo). Interviews were conducted in Lingala, Kiswahili, and occasionally French, audio-recorded with participants’ permissions and subsequently transcribed and translated into English by professional Congolese translators. The interviews were conducted privately, and no identifying information was collected. All interviewees gave age-appropriately informed verbal consent to participate and have the anonymised results published. After mothers had participated in the study themselves and understood the nature of the research, the participation of their PKFC was discussed and parental consent recorded. The study protocol was approved by the institutional review boards of Queen’s University (6019042) and the University of Birmingham (ERN_18-0083; ERN_17-1715), as well as the Congolese National Committee of Health Ethics (CNES 001/DP-SK/119PM/2018) (see Wagner et al. 2020 for more information on study design and implementation).

In the first step of data analysis, interviews were classified into three categories: those illustrating low social status, average social status, and high social status. Low social status was attributed when participants referenced negative stereotyping, stigma and social exclusion. A separate analysis of stigma and its most dominant causes can be found in the study of Wagner et al. (2020). High social status was attributed when participants referenced being considered special, admired and treated preferentially. We have chosen to focus on social status as a meaningful construct and dimension of social identity that illustrates individual and social perceptions (Cheon et al. 2020; Oldmeadow and Fiske 2010). Originating from this three-tier classification, content analysis was used to identify frequently and infrequently endorsed factors contributing to social status. The resulting codes reflect the social standing of PKFC, evaluated by themselves and their mothers. Deductive codes included topics from the survey, such as participants’ living circumstances and interpersonal relationships with family and community. Inductive codes included cultural constructions and social discourse regarding the local norms that shaped participants’ social interactions. After compiling a comprehensive list of codes, their presence and absence in each group of interviews was assessed and compared.

While several factors played into PKFC’s experience of status (e.g., family structure, their context of conception—not the specific subject of this study—socio-economic status and racial background), individual factors alone could not explain the range of status differences. Based on this observation, we looked at the intersection of several factors and found that PKFC’s race and economic means influenced social identity in a unique way. In the following sections, we will show that whether PKFC have average, higher or lower social status than their peers is impacted by racial group variations and financial disparities.

## Findings and Interpretations

**Fig. 1:**
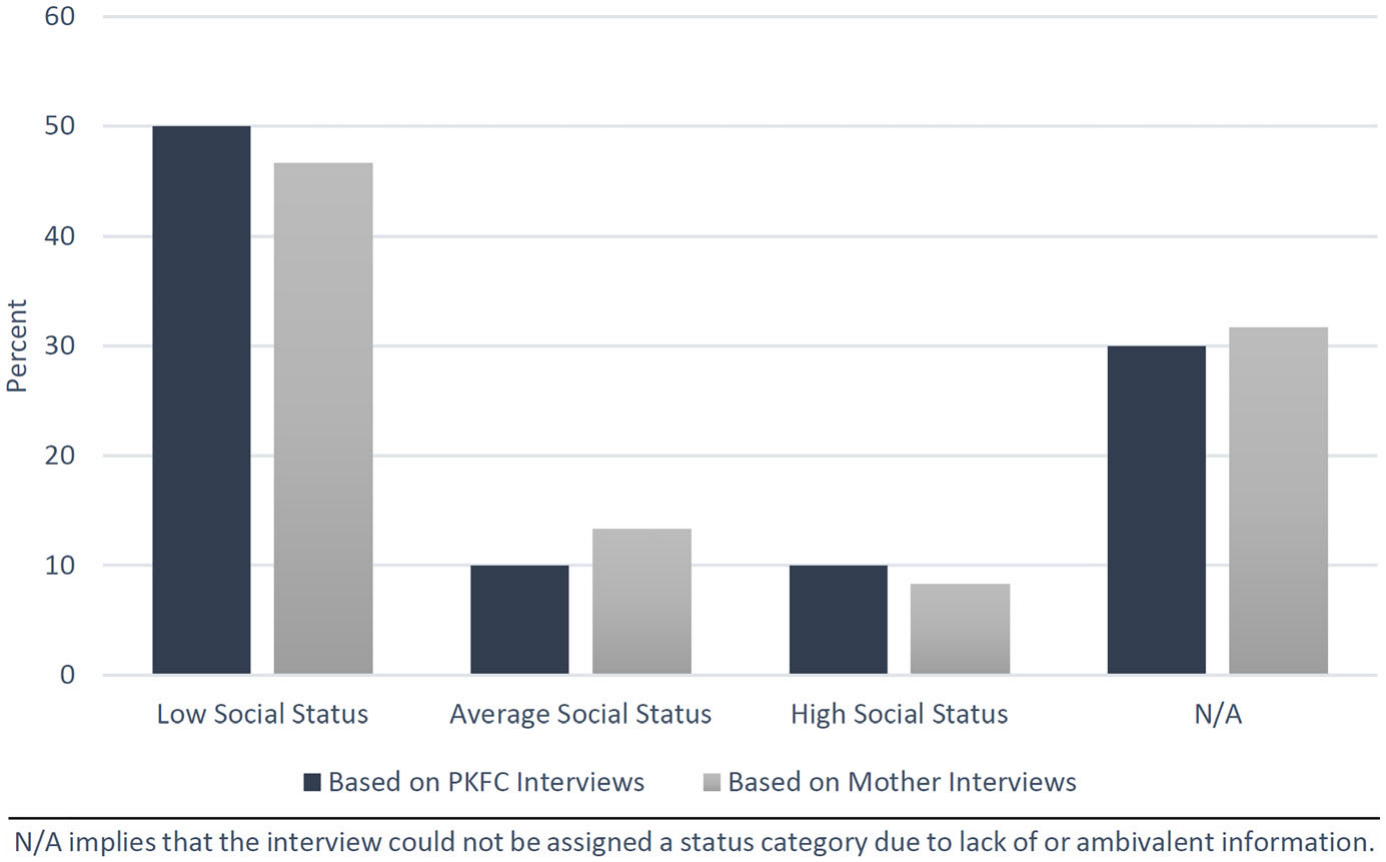
Observed distribution of social status in PKFC

Where peacekeeper fathers absolve themselves of their paternal responsibility, their children’s social identity tends to be characterised by increased economic and social volatility, translating into low social status (Wagner et al. 2020; Vahedi et al. 2020). Drawing on both mothers’ and PKFC’s interviews, we found that most PKFC experienced severe ‘othering’—a central mechanism of social exclusion that has widely been discussed in literature on DRC wartime sexual violence (e.g. Koos and Summer 2019). Exposure to stigma and ostracization represented a formative experience in the childhood and adolescence of many PKFC and complicated their identity development (Wagner et al. 2020). However, in spite of the overwhelming impression that PKFC faced high levels of stigma, a minority of PKFC recounted having no experience with harmful stereotyping and societal shame. Based on our analysis, three of the 35 PKFC did not perceive themselves to have deviating social status and described acceptance by family and community; an additional three implied having higher social status than their peers. The latter described that they were being treated preferentially and regarded highly by family members, friends and teachers. In line with this, mothers occasionally detailed that their child elevated their family’s social standing. Thus, the findings highlight a spectrum of social identities with social status, occasionally increasing rather than decreasing (Table 1; Fig. 1).

**Table 1:** Low vs high social status

	Low social status	High social status
Social status of PKFC (Evaluated by mothers)	“The neighbours don’t love her. My friends were even bringing different kinds of poisons in order to kill her. They constantly make fun of her saying that she is the daughter of a white. Because of this, she only stays here in the compound; she is ashamed.” (Mother)	“My child is a star. He is loved everywhere. Some people don’t even accept me as his mother. My relatives want to live with him and pay his school fees, but he prefers to stay with me because he loves me so much. I think my child will be somebody of great importance.” (Mother)
Social mobility of mothers (Evaluated by mothers)	“My family rejected me. They no longer speak to me. ‘No one sent you to make a baby of that skin colour’, that’s basically what I always hear from them.” (Mother)	“My friends and family members were happy with me because of this baby. In short, I was like a star… At that time even most MONUC agents were happy with me.” (Mother)
Social status of PKFC (Evaluated by PKFC)	“My neighbours hate me and say that I have to look for my father. They backbite me whenever I attempt to talk. My community laughs at me[…] I am worried a lot; I am not stable enough to live such a life.” (PKFC)	“My mother loves me so much. She doesn’t like me to leave and go somewhere else. Among her children, I am the only one who has a high level of education[…] I hope to become an important person in the future.” (PKFC)

Arising out of these experiences, we will discuss the influence racial identification and poverty have on status differences. The first section addresses race as an indicator of social status and examines race-related cultural and societal perceptions. The second section addresses poverty as an indicator of social status and demonstrates the stigmatizing effects of low economic means. The final section connects the two and illustrates how the nexus of race and poverty shapes mothers’ social mobility and youth’s social identity. Representative quotes were chosen based on contextual relevance to illustrate key themes.

### First Axis: Race

Mothers who recalled the nationality of peacekeeper fathers named Tanzania, Uruguay, Morocco, Nigeria, South Africa, Nepal, Bangladesh, Sudan, DRC, Benin, Malawi, Senegal, and Guatemala as countries of origin. Thus, the intra-categorical complexity of race for PKFC posits a range of youth who are dark-skinned (due to Congolese or other African descent) and fair-skinned (due to North African, Asian or Latin American descent). Since their individual appearance resembles their unique racial heritage, organizing PKFC as a racial category is difficult. We found that in the local context, race was thought about in binary lines but included ‘skin tone bias’ and the reality of ‘colourism’—PKFC being treated differently based on whether they had a lighter or darker skin (Hunter 2005). Although one mother used the term “mulatto” to refer to her child, most participants applied the racial/pan-ethnic terms “black” and “white” and then added ethnic/national terms to those lines, stating that, for instance, “the white is [was] from Nepal”. Thus, rather than being considered of mixed race or identified due to their dual racial background, PKFC were initially labelled black or white.

There are so many children here who were forgotten. Black and white children. (Mother)

In line with how mixed race typically defaults individuals in predominantly white countries to the non-white category and individuals in predominantly black countries to the non-black category (Rost-Banik 2020), PKFC whose physical appearance differed from the local norm usually identified as white. Thus, the term ‘white’ was most consistently used to describe mixed-race youth fathered by UN peacekeepers from countries outside central Africa. However, it was sometimes also applied to individuals fathered by UN staff from surrounding countries or more urban areas in DRC. Echoing that there is “no monolithic version of whiteness” (Clements and Mason 2020:485), we will refer to ‘whiteness’ as a socially constructed racialized identity that, even within the same cultural context, holds different connotations, some of which do not refer to biological race (Table 2).

**Table 2:** Identity labels and categories for PKFC

Racial identity/identification	“My friends are black, but I am white.” (PKFC) “He is black like them.” (Mother)
Ethnic identity/identification	“I am white. The white is from Morocco”. (PKFC) “I take my child as Tanzanian. Her name was given to her by her father[…] When I think about the future of my child, I picture her in Tanzania.” (Mother)
Clan identity	“Some people know about the child’s backgrounds; others don’t want to think about it because we can’t be certain of his tribe[…] We are worried what will become of our children since they will want to know their tribe and we have nothing to tell them.” (Mother)
Nationality	“He always says that he is Beninese. In school, they interview children for their nationality. The child says that he is Beninese. My father is Beninese; therefore, I am Beninese. He is old enough to reply to these questions.” (Mother)
Citizenship	“I take him as Congolese, but I haven’t registered him with the state.” (Mother)

Based on our analysis, PKFC’s racial background accentuates their social status. Participants’ extensive referencing to race and racial stigma suggests sensitivity to racial identity and group differences for PKFC of all ages. Physical features that evidently identify PKFC with their fathers’ lineage made them an easier target for racial stigmatisation and conveyed more potential for societal rejection. PKFC reported being singled out or verbally attacked on the grounds of being white or visibly linked to their foreign fathers. Mothers who had witnessed their children being socially excluded due to their white identification also reported that being “see[n] with a white baby” affected their own social status.

They call him Muzungu or white boy. They stigmatise him because I, the mother, am black, but the child looks different from my other black children as far as complexion and hair are concerned. They simply say that he is a white, not a black child[…] When people see my child, they often wonder how a black woman can deliver a white child. They say that the child is white so he should go to his father. (Mother)

**Fig. 2:**
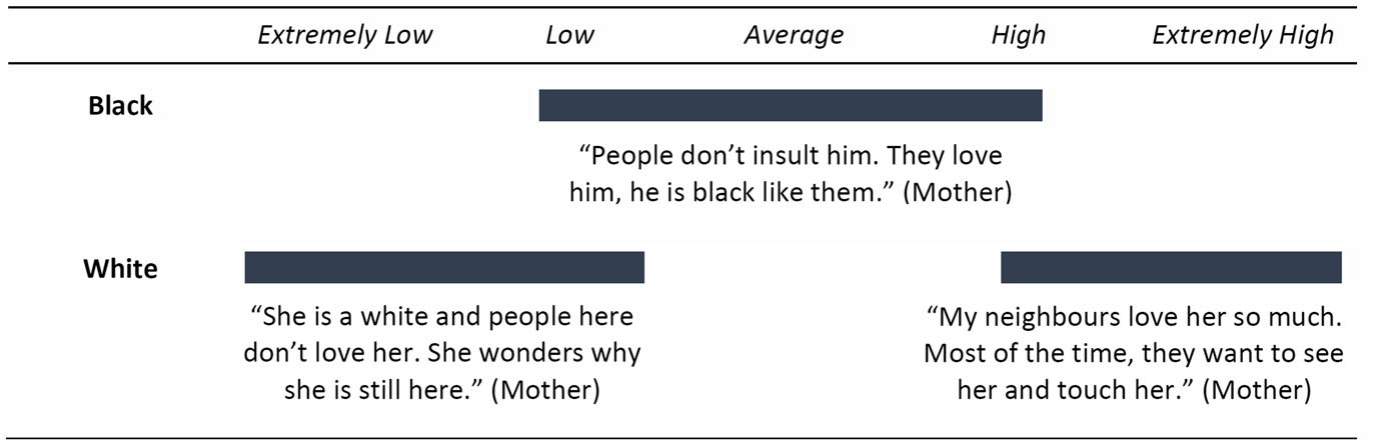
Observed variance in *social status* based on **race**

Overall, black PKFC were less often confronted with stereotyping and prejudice; likely because their group membership as PKFC was less salient and they assimilated more easily into the local culture. This suggests that ethnically mixed youth experience less social status deviation if they do not stand out as biracial and confirms that the value placed on racial identity is higher for those with obvious genetic markers (Turner and Brown 2007; Rogers et al. 2012). While for black PKFC no significant within-group variation was observed (black PKFC predominantly shared average to low social status), the social standing of white PKFC varied widely, from extremely low to extremely high. Despite the large majority of white PKFC experiencing negative stereotyping, some were “loved and admired because of her [their] skin colour” (Fig. 2).

PKFC discussed their racial identity mostly in terms of their appearance and related group differences. Some PKFC referred to other mixed-race youth as “kids like me” (see Meltzoff 2013 for “like-me” social categorization). Since young people’s racial identity becomes increasingly salient with heightened awareness of group boundaries, biracial identity scholarship emphasises that racial diversity helps biracial youth navigate identity challenges (Rumbaut 1994). However, our participants did not live in racially diverse communities, and had little exposure to other racial groups, as the following example shows.

She doesn’t know her father. She only saw the chief of her father once when he came to visit the children who are left by MONUSCO agents for support issues. When she realised that he was white, she decided that he was her father. (Mother)

According to social identity theory, the lack of racial reference groups (‘others like me’) might pose stress on PKFC’s sense of self. While it is assumed that many mixed-race children have a conflicting racial identity, children fathered by peacekeeping personnel are also confronted with the uncertainty of their lineages that deny them making sense of their ancestry. This has been found to push some PKFC to engage in wishful thinking and imagine an idealised version of family and identity (Wagner et al. 2022). In patrilineal African societies, social status (e.g., rights, obligations, privileges) is linked to belonging to a clan that is confirmed in children’s names and determined through their paternal family (Lee 2017). Mothers indicated that the absence of clan privilege and inability to trace their fathers’ tribe left PKFC feeling uncertain of their role within communities (see Table 2). Family has a critical role in helping children cope with identity stressors (Crawford and Alaggia 2008), yet PKFC often received limited care and attention from family and were left alone with the task of resolving their identity (Wagner et al. 2022). When self-esteem is at substantial risk, biracial individuals might distance themselves from one racial group or try to reduce dissonance and the climate of conflict otherwise (Rockquemore, Brunsma and Delgado 2009). We found that some PKFC developed a sense of belonging to their fathers’ high-status minority group and, despite their absence, sought contact points with their fathers’ culture.

My boy is now nine years old. I told him that the has the name of his father and that he is Nigerian, he is very happy with that. He is crazy about his father. He said one day he will go to Nigeria. He feels alright, but he asks me a lot of questions when he sees UN cars. He asks me if his father was working with people driving in such cars. (Mother)

Interestingly, families and communities placed a number of role expectations upon white PKFC that seem to originate from the historical legacy of colonialism and a social organisation that privileges whiteness. The data holds strong evidence that there is a distinct cultural understanding of whiteness that predicts fair-skinned PKFC to be “different from local kids in every way possible”.

She behaves like white people. Whenever I buy something for her siblings, she wants to have it. I often meet with other women who are in the same situation. Whenever we meet, we cry together because we think about how hard it is to raise that kind of children. White children and black children are very different; the white one wants to sleep well, eat well and live in a given comfort. That lifestyle is difficult to provide. (Mother)

According to our data, mothers viewed white children as distinct from black children in behavioural characteristics. Reportedly, white youth required more care and attention than black youth, were “aggressive”, “egoistic”, or wanted to “show off”. Mothers mentioned that due to their white consciousness, PKFC were “too demanding” and had “luxurious” preferences regarding food, clothing, or skin care. With respect to food choices, many mentioned PKFC asking for “western” food like fries, doughnuts, or sweets instead of rice, bread, and vegetables. This led mothers to conclude that it was “seriously hard to raise a child of this [white] skin colour” (Table 3).

**Table 3:** Stereotypes for white PKFC

Food	“He doesn’t eat the same food as others. He doesn’t like fufu or cassava-bread, rice sometimes. He prefers things like pancakes, drinking juice. He gets angry when we can’t find the food of his choice. He doesn’t insult us, but he shows that he is furious to death. When he gets the food he likes, he cools down. His temper is like sitting on a volcano.” (Mother)
Clothes	“The child doesn’t really want to wear common or more modest outfits; he enjoys fashionable clothes.” (Mother)
Skincare	“It’s not easy to pay for medication and also the ‘Bazungu’- special white race body lotion that he needs. I was lately in Bukavu to find one, but I was unable to afford it. I went to a supermarket called ‘la beauté’ and to an Indian shop in Bukavu, but in both places, it was too expensive. It costs 27 dollars.” (Mother)
Lifestyle	“Sometimes I don’t understand him. He wants to live a luxurious life; he wants to be smart and have sophisticated things.” (Mother)
Mood	“Each child has got her father, both are foreigners[…] They are very shy and don’t play games. Others say children who are two years old can laugh, but this one instead, she cries. She is aggressive and violent smacking towards others.” (Mother)
Behaviour	“She behaves differently to other children. This child is so agitated. She is aggressive, and she likes to fight. Her peers push her to fight. She is rude and aggressive.” (Mother)

The adopted characteristics of whiteness appear to be perpetuated by cultural stereotypes, media portrayals and the socio-historical understanding of whiteness. This shows that in the local context, whiteness is highly racialised. PKFC’s families and communities create two social categories for PKFC and express the attributes and societal worth of their assigned group membership. PKFC’s ‘degree of blackness’ thus significantly impacts how central race is to their existence and on whether racial discrimination presents a daily concern.

### Second Axis: Poverty

The DRC has one of the highest rates of extreme poverty globally with exceedingly high levels of food insecurity, low life expectancy, and poor access to health care and education (Azzarri and Signorelli 2020; UN Development Programme 2019). Recruiting participants from impoverished neighbourhoods meant that PKFC were likely to live in economically strained households. Fatherlessness and the resulting living circumstances further diminished their economic security. Consequently, the intra-categorical complexity of poverty for PKFC posits a range of youth from socio-economic circumstances that are similar or lower to those of most families in eastern DRC, with occasional outliers of higher socio-economic means. Poverty status was assessed based on food insecurity, inadequate housing, poor health conditions, occupational status, and educational attainment. While most PKFC recounted income indicators and access to resources that placed them below the average (operationally defined as extensive referring to unmet basic needs), a handful of PKFC were living above this threshold. We will refer to those participants as ‘relatively wealthy’.

About me? Let me tell you that I never go to school. I have no good clothes. When I think of the deep poverty I’m in, I feel much despair. I’m dressed poorly. I have no body lotion, not even the local palm oil or soap to wash my face. I have no food. My life is nonsense. Hard life conditions. It’s too harsh, too bad actually. Nothing changes for the good ever. My family goes through much pain to find the amount of food we need daily[…] On many occasions, we go to bed without having eaten anything. I have no shoes, not flip-flops either. You can see that what I am wearing is completely torn apart. (PKFC)

As shown previously (Wagner et al. 2020), the social challenges of PKFC are often caused and compounded by poverty. Mothers mentioned that their lack of financial means to cover their children’s basic needs lowered their social status which in turn made them lose the support of family and friends who did not want to carry the burden of association with them. In this way, the mutual reinforcement of marginalisation and economic hardship caused a spiral of more extreme stigmatisation and poverty. Several mothers pointed out that their “story is [was] just one example” of how raising a PKFC caused “miserable life conditions”.

We are carrying a heavy burden raising abandoned children without any means. The other mothers – there are so many – are also sitting on a mountain of problems. They exercise no activity; they do nothing and are in very critical situations. We have all become like street children. (Mother)

Mothers’ social mobility varied with the level of support received from former partners. Many perceived that the public knowledge of their partner's neglect, evidenced in their apparent struggle and economic disadvantage, contributed to their negative status and reflected poorly on their social network and community. Relatedly, mothers who saw their PKFC as a reminder of their own victimisation developed ambivalent feelings towards them that sometimes resulted in child abuse or neglect. Occasionally, mothers spoke about initial improvements in social status due to the PKFC or explained that they had been met with jealousy and envy during the pregnancy but that this changed once the child was born and the fathers absolved themselves of their responsibilities. One mother who initially received noteworthy support from the PKFC’s father detailed that her family began struggling with social stigma since that support ceased.

People laugh at me because of my situation these days. Formerly, when the man was still alive, the kids were going to good schools, but now that the situation has changed, the topic became subject of their gossip. They say my children are no longer looking good. (Mother)

**Fig. 3:**
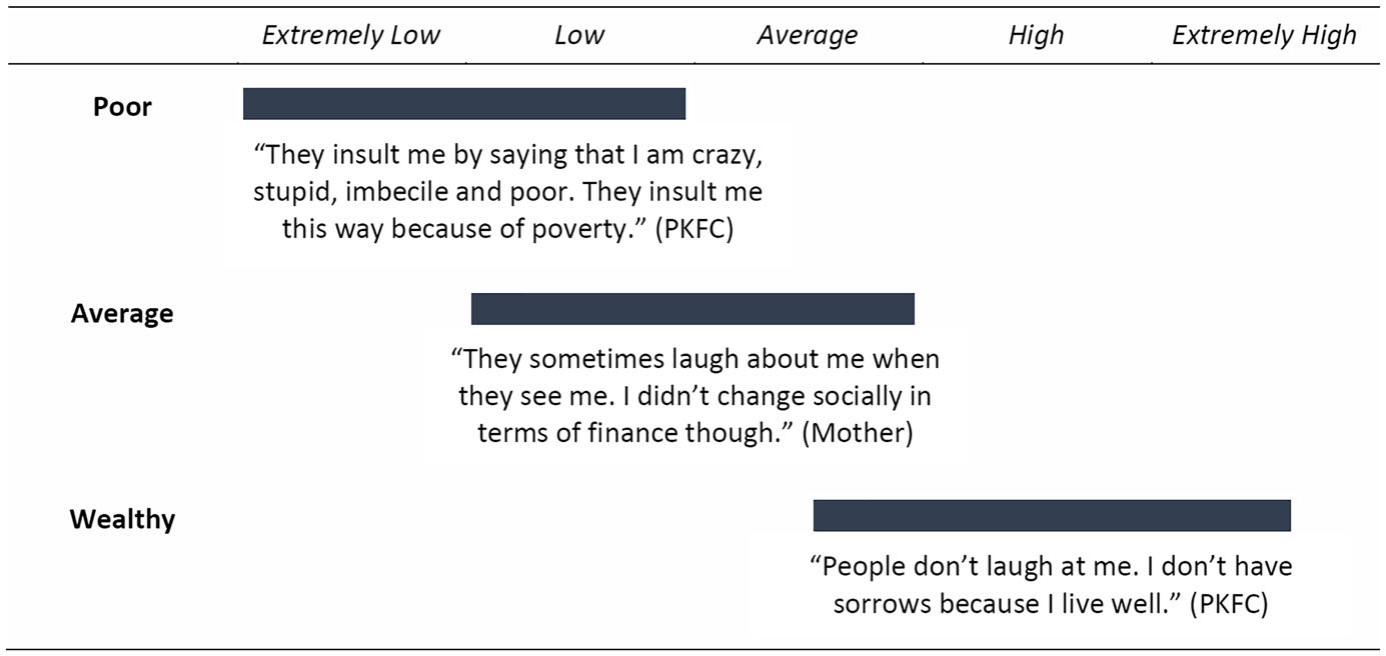
Observed variance in *social status* based on **poverty**

We found that high levels of stigma and discriminatory treatment were reported by individuals who suffered high levels of economic concerns and perceived threats to resources while individuals who reported low levels of stigma articulated fewer economic concerns and struggles to survive because of poverty. Participants who felt broadly accepted, insinuated that their situation was exceptional because of their high living standard, implying that most others did not benefit from such circumstances. Thus, there seems to be a clear (and largely linear) relationship between participants’ level of poverty/support received and their social status (Fig. 3).

### The Intersection of Race and Poverty

Among research about the relationship between racial identity and poverty, a particular knowledge gap relates to this intersection in the post-colonial settings of the global South. Based on the experiences of PKFC, we will discuss how financial disparities interact with racial group memberships and contribute to a privileged or devalued social identity.

Table 4 introduces three patterns of interaction that were observed: (a) an average level of poverty translates into low social status for white PKFC and average social status for black PKFC, (b) the interaction effect of poverty and race is larger for PKFC who are considered white than PKFC who are considered black, (c) for white PKFC variance in poverty might lead to one of two extremes.

What does this mean? The effects of poverty on social status are amplified by white racial identification. This showed particularly in the sample outliers—participants whose social status was extremely high or low (Table 4).

**Table 4:** Observed interaction of race and poverty

Poverty × Race	White	Black
Poor	Extremely low social status	Low social status
Average	Low social status	Average social status
Wealthy	Extremely high social status	High social status

Poverty status appears to be able to explain the considerable within-group variation in the social status of PKFC who are perceived as white. We argue that this is due to a social comparison between the role assigned to white youth (introduced in Table 3) and their ability to adhere to their attributed group membership (Hechter and Opp 2001). In other words, the social status of PKFC depends on their ability to enact the local cultural norms of their communities (Chandler and Wiborg 2020). As discussed in the section on race, host state communities in DRC expect white youth to abide by a certain ‘norm of whiteness’ that is informed by a stereotypical representation of the white elite. White youth were considered to live “in a given comfort” and were predicted to “become important people” who would attain positions of power, become “ministers, doctors and so on”. As discussed in the section on poverty, the financial reality of most PKFC was characterized by extreme hardship and deprivation, lack of support and family connections. Thus, securing a “bright future” along the lines of economic and educational achievement stands in stark contrast with the lived reality of most PKFC (Vahedi et al. 2020). For the large majority of participants, the expectations that the Congolese society sets for white youth were highly incompatible with their social and financial realities. Scarce financial means made day to day expenses difficult and did not allow for a luxurious lifestyle (Table 5).

**Table 5:** Aspired vs actual living standard

	Aspired living standard	Actual living standard
By PKFC	“I hope to become an important person in the future.” (PKFC)	“In order for me to be like him, I must go to school, but it is very difficult for me to go to school.” (PKFC)
By mothers	“I pray that God keeps my child safe as he can assist me in the future. A white child can be helpful in that way.” (Mother)	“My child is deprived of everything: lotion, soap, clothes, and food. Nobody can really believe that this is the child of a MONUSCO agent, it doesn’t have a decent living standard.” (Mother)

Not being able to meet the expected social norms aggravated stigma related to poverty and reversed racial privilege through sanctions (Hechter and Opp 2001). A derogatory term for PKFC in DRC is “Muzungu aliye homba” [white child gone bankrupt] which conveys traces of gloating about failed white privilege. The common term in central Africa, “Muzungu” [white/European] is used as an indicator of race but is also linked to economic means and a higher living standard. Thus, the cultural perception of “Muzungus” is that of power and wealth, an implication of whiteness shaped by historical racial inequality (Jourdan 2013). Similar to racial slurs like “white trash” or “cracker” (Wray 2006), the adaptation “Muzungu aliye homba” indicates that PKFC defy the white-racial frame of reference by being poor and powerless despite their association with racial privilege. Hence, the term illustrates their non-adherence with the ideology and practices of the white elite. Poor PKFC being reprimanded for the discrepancy between their expected and actual living standard is in line with the ‘status incongruity hypothesis’ that predicts backlash towards people who behave incongruous to behaviour stereotypes associated with their social group membership (Rudman et al. 2012). By contradicting the established racial logic, poor PKFC form a racial ‘other’ and are denied the structural benefits of whiteness (Cushing-Leubner 2020). To describe the implication of this intersection, we introduce the term ‘white-poverty stigma’.

The others tease him saying he’s a white South-African walking around like a poor local child. They call him a ‘South-African son – a boy with an unknown father’. Sometimes when others tease or mock him, he gets very angry and he falls out with them. (Mother)

Drawing on the work of others (e.g. Cooley et al. 2019), we suggest that the mechanism behind white-poverty stigma is the social evaluation that white individuals have failed to take advantage of their racial privilege. In addition to the external sanctions of status incongruity, PKFC might wrestle with a threatened self-concept and experience a sense of inferiority towards their absent fathers’ high-status minority group (see Steele and Aronson 1995 “stereotype threat”). The data holds an indication of harmful consequences for self-regard and psychological well-being when PKFC were not able to manage the demands of their group membership. For instance, being neglected the social, political and economic privileges of their fathers might have caused PKFC to act out and explain the behavioural problems mothers observed in their children (see Table 3).

Interestingly, the few PKFC in the sample who were categorized as white and relatively wealthy seemed to derive social benefit from their intersecting group memberships. It appears that PKFC who receive support from fathers or are from an otherwise more privileged background are rewarded for enacting their presumed privilege (Chandler and Wiborg 2020). Instead of battling with the social sanctions attached to violating stereotype norms, they were found to receive financial contributions that elevated their social status further (see Table 1).

Many people like him and give him 5 dollars or 10, especially when we walk around the airport. People are happy to call him a white boy. (Mother)

While financial resource flow within Congolese family and community networks is not unusual (De Herdt 2007), we perceived this to be related to a preconception according to which white PKFC were to become gatekeepers to economic relief. As the following excerpt shows, families hoped that white youth could help them move towards a better future.

The child indeed is white, so he might be my salvation one day. (Mother)

The societal expectation for PKFC to play a role in the local economy (whether adhered to or not) resembles elements of ‘white saviour discourse’ (Cole 2012; L’Anson and Pfeifer 2013); a narrative that might be encouraged by PKFC’s link to UN peacekeeping and the white dominated aid industry that is in control of desirable resources.

Based on those social expectations, our data suggests that lack of access to resources and essential life domains like education affects the social status of PKFC more than it would affect other youth. This is especially the case for white PKFC who sit in tension with their assumed white privilege (represented by their white elite fathers) and their black, natal realities (represented by their poor local mothers). While the social status of black PKFC is primarily driven by their level of poverty, that of white PKFC is compounded by the status expectations for white youth.

## Limitations

The present study offers a first account of the essence of PKFC’s relationships with society, rather than exact representations of race or patterns of child poverty. Our results inform the experiences of a relatively small number of PKFC and are not representative of PKFC globally. In order to address PKFC’s situation ethically, the research focused on the context in which children lived and engaged them with their own living environment. Words like ‘poverty’ or ‘poor’ were not used by the interviewers and no quantitative information on family income was collected. Similarly, PKFC were not asked to systematically reflect on their racial identity but instead were left to bring up race-related issues themselves. Consequently, not every interview could be attributed a respective social category and the related interpretations should not be seen as absolute. To capture race and poverty as truly intersectional, explicit questions about what it means to be ‘poor’ and ‘white’ need to be asked. Whether assessing these concepts more directly in research with children is possible is a question of ethical considerations.

The authors discuss the upbringing of youth aged 6 to 20, yet the research did not systematically differentiate between the experiences of children and adolescents and did not follow PKFC over time. Instead, a cross-sectional analysis of their situations was undertaken and trends and patterns for PKFC of different age groups observed. More controlled study settings and longitudinal research identifying similarities and differences in the experiences of PKFC over time, including later stages of life, are necessary to provide a more comprehensive picture of their development. We acknowledge biases inherent in our position as non-Congolese adult researchers.

## Strengths

The strength of the current research lies in the multidisciplinary collaboration of scholars and civil society organizations and its interdisciplinary and intersectoral approach. Due to the non-invasive interview style and support of local experts, children as young as six were included in the research. To our knowledge, this is the first study to focus on the racial background of PKFC and reflect on them as a racially heterogeneous population. Drawing on 95 qualitative interviews, we contribute novel information to the currently emerging database regarding the life courses of these youth. Employing the perspectives of both PKFC and mothers triangulated findings about social identity and identification and enabled a nuanced interpretation of their experiences. Historically informed and contextualized, our analysis bridges the individual accounts of PKFC with broader discussions about race in host state Congolese communities. The study advances scholarship in the arts and social sciences regarding the perspectives of children born of war and informs psychological discourse regarding racial awareness and identity in biracial youth in central Africa. While our results are specific to PKFC and the post-colonial setting of DRC, it is likely that white-poverty stigma can be observed in impoverished situations in other populations. Their interdisciplinary and intersectoral approach makes our results not only of direct interest to the UN but also to academics, NGOs, and human rights organizations working to safeguard vulnerable youth in conflict.

## Conclusion and Recommendations

This is the first study to empirically explore the racial identity and differences of PKFC. Discussing social identity as the qualitative outcome of different group memberships suggests that the psychological experience of race is interrelated with economic dimensions. Our results show that whether PKFC are afforded unique opportunities depends on the relationship between their expected and actual living standard. In showing how PKFC challenge the prevailing racial order, we make the contextual terms and historic moment explicit in which PKFC live and thus provide new context for targeted interventions. Based on the testimonies shared, we make two recommendations to advance meaningful policy solutions:

First, while in theory the UN has set out a support system for PKFC, very few (if any) PKFC are regularly receiving child support or other UN subsidies (Blau 2016; Office of Internal Oversight Services 2015). The current study shows that PKFC’s financial hardship is connected to other areas and highlights that programs designed to alleviate poverty might transform more than one strand of disadvantage. Thus, the knowledge of white-poverty stigma reinforces the importance of addressing poverty. Reparation payments may help reverse social sanctions and work towards reconciling PKFC’s relationships, for instance, through repaying family debt (Adams 2011).

Second, participants’ lack of racial reference groups and contact with ‘others like them’ complicates the resolution of their racial identity. Most youth reported knowing others with similar procreation backgrounds, yet few were connecting with them regularly. Forming support networks with other PKFC and tackling identity issues as a group might bring relief from some of the burdens addressed in this study. Since peer support activities are a low-cost approach that has empowered children born of war in other contexts (Stewart 2015), building relationships with peers might have protective effects against negative group-related experiences and mistreatment.

### Areas for Future Research

In this study, we found the intersection of race and poverty to be an observable aspect and indicator of PKFC’s social identity. However, as is the nature of an intersectional analysis, this is not an exhaustive list of factors determining how PKFC are perceived socially. We therefore encourage future research to consider other influences like PKFC’s context of conception (consensual, exploitative, or abusive sexual relations) family structure (nuclear, single-parent, extended family) and gender differentials (male, female, non-binary) in their impact on social identity.

In placing the categories race and poverty at the center of the intersectional analysis, we have distanced our work from the origins of intersectionality and the seminal work of black feminists who developed the concept with gender as an inherent component. In doing so, we do not imply that gender is unimportant, but we respond to the concerns of those interviewed for the present study. Moreover, we argued that intersections with gender will become more clearly reflected in older PKFC and should therefore be explored in studies with young adults. Based on the interviews conducted with adolescents, our findings provide a number of starting points for future research on gender differences in the experiences of PKFC. We found an indication that fatherlessness and the related socio-economic disadvantage may be more severe for boy PKFC than girl PKFC. While in traditional African societies, characteristics for women are centred around their identities as mothers and caregivers, men are perceived to be providers, income earners and homeowners (Gilliard 2010; Puechguirbal 2003). The limited access of male PKFC to education, family inheritance and social networks limits their chances of fulfilling their anticipated gender role; a difficulty that is amplified by the pressure they experience to follow in their father’s footsteps and abide by the perceived ‘norms of whiteness’. Male PKFC disclosed their intention to “become an important person in the future” or grow up to “be like him [their fathers]”; thus, they may be looking to express their masculinity through attaining high status positions. If, in reality, they are confronted with a lack of prospects and opportunities, male youth may face additional stigma. In the DRC, boys who see their male privilege undermined have further been found to have a higher likelihood of being recruited by Congolese military and rebel groups, increasing protection risks for male PKFC (Bastick, Grimm, and Kunz 2007).

While impacted less significantly in their traditional contribution to society, the low social status of female PKFC may limit their bride value; and hence, their ability to secure a dowry and resources. In addition, gender differences in resource allocation within Congolese households historically limits investment in girl’s education and thus, families with insufficient funds to send all children to school will likely prevent female PKFC from being educated, denying them vocational skills and self-sufficiency (Meger 2010; Shapiro and Oleko Tambashe 2001). These observations echo the experiences of children born of war in other contexts in SSA with similar gender role expectations (Stewart 2015). How exactly gender impacts on the social status of PKFC and interacts with other identity-forming characteristics remains to be established through more targeted research.

## Data Availability

The datasets used during the current study are available from the corresponding author on reasonable request.
